# Two cases of strangulated bowel obstruction due to exposed vessel and nerve after laparoscopic and robot-assisted lateral lymph node dissection (LLND) for rectal cancer

**DOI:** 10.1186/s40792-024-01889-8

**Published:** 2024-04-15

**Authors:** Ryota Fujiwara, Masaaki Yano, Makoto Matsumoto, Tomoaki Higashihara, Shimpei Tsudaka, Shinsuke Hashida, Shuji Ichihara, Hiroki Otani

**Affiliations:** Department of Gastroenterology and General Surgery, Kagawa Prefectural Central Hospital, 1-2-1 Asahimachi, Takamatsu, Kagawa 760-8557 Japan

**Keywords:** Internal hernia, Obturator nerve, Lateral lymph node dissection

## Abstract

**Background:**

The majority of small bowel obstructions (SBO) are caused by adhesion due to abdominal surgery. Internal hernias, a very rare cause of SBO, can arise from exposed blood vessels and nerves during pelvic lymphadenectomy (PL). In this report, we present two cases of SBO following laparoscopic and robot-assisted lateral lymph node dissection (LLND) for rectal cancer, one case each, of which obstructions were attributed to the exposure of blood vessels and nerves during the procedures.

**Case presentation:**

Case 1: A 68-year-old man underwent laparoscopic perineal rectal amputation and LLND for rectal cancer. Four years and three months after surgery, he visited to the emergency room with a chief complaint of left groin pain. Computed tomography (CT) revealed a closed-loop in the left pelvic cavity. We performed an open surgery to find that the small intestine was fitted into the gap between the left obturator nerve and the left pelvic wall, which was exposed by LLND. The intestine was not resected because coloration and peristalsis of the intestine improved after the hernia was released. The obturator nerve was preserved. Case 2: A 57-year-old man underwent a robot-assisted rectal amputation with LLND for rectal cancer. Eight months after surgery, he presented to the emergency room with a complaint of abdominal pain. CT revealed a closed-loop in the right pelvic cavity, and he underwent a laparoscopic surgery with a diagnosis of strangulated SBO. The small intestine was strangulated by an internal hernia caused by the right umbilical arterial cord, which was exposed by LLND. The incarcerated small intestine was released from the gap between the umbilical arterial cord and the pelvic wall. No bowel resection was performed. The umbilical arterial cord causing the internal hernia was resected.

**Conclusion:**

Although strangulated SBO due to an exposed intestinal cord after PL has been a rare condition to date, it is crucial for surgeons to keep this condition in mind.

## Background

The majority of small bowel obstructions (SBO) are caused by adhesion due to abdominal surgery. Internal hernias, a very rare cause of SBO, can arise from exposed blood vessels and nerves during pelvic lymphadenectomy (PL). In this report, we present one case each of strangulated bowel obstruction caused by herniation of obturator nerve after laparoscopic lateral lymph node dissection (LLND) for rectal cancer and by herniation of the umbilical arterial cord after robot-assisted LLND, with a review of the literature.

## Case presentation

**Fig. 1:**
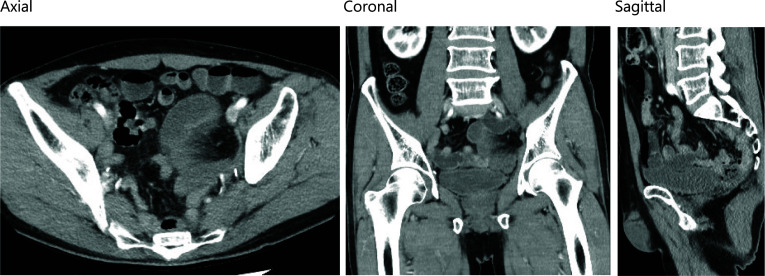
Contrast-enhanced CT scan of the case 1 showed closed-loop in the left pelvic cavity (left: axial, center: coronal, right: sagittal)

Case1: A 68-year-old man visited our emergency room for the complaint of subacute left groin pain, which gradually worsened. Four years four months prior, he had undergone a laparoscopic abdominoperineal rectal amputation with LLND for rectal cancer. The LLND was a prophylactic dissection that spared the autonomic nervous system and the internal iliac vasculature (upper and lower bladder vessels). Seprafilm was placed postoperatively in the pelvis and just below the midline wound as an anti-adhesive. After surgery, he received standard systemic adjuvant chemotherapy. No recurrent sign was observed. Contrast-enhanced computed tomography (CT) images revealed that SBO with a closed-loop had occurred in the left pelvic cavity (Fig. 1). Although the edema of the mesentery was mild and there appeared to be a mild contrast effect on the small bowel wall, there was a possibility of intestinal congestion. He had no abdominal pain, and he had tenderness pain in his left groin and medial part of the thigh. The pain was more pronounced in the left medial thigh than in the abdominal symptom, which was atypical for a strangulated bowel obstruction. However, the patient's symptoms did not improve, and strangulated bowel obstruction was suspected from the imaging findings.

**Fig. 2:**
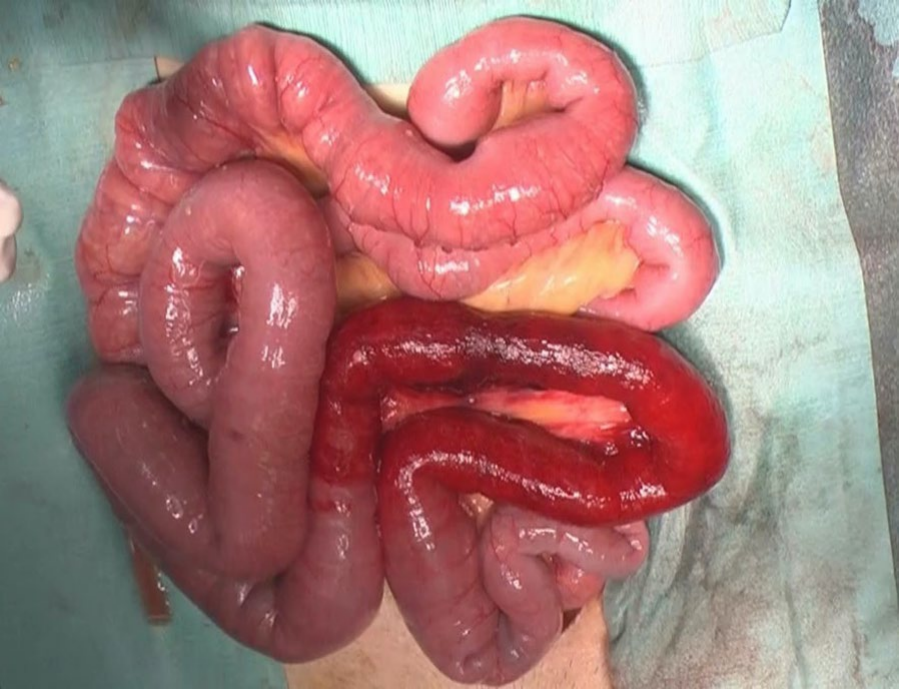
Small intestine after release of strangulation in case1

Therefore, we performed emergent open surgery and found the strangulated small bowel with bloody ascites in the left pelvis. The small bowel did not adhere to itself and to the abdominal wall. Approximately 30 cm of the small intestine, located 60 cm proximal to the terminal ileum, was found to be herniated into the gap between the left obturator nerve and the retroperitoneum left pelvic side wall, exhibiting signs of ischemic discoloration. We easily released the incarcerated loop from the hernial orifice. As the color and peristalsis of the ileum improved, intestinal resection was not performed (Fig. 2). On the other hand, there was no tissue available to close the gap caused by the exposed left obturator nerve. Considering the risk of postoperative motor dysfunction if excised, no measures were taken to prevent herniation. He was discharged with no important complication on the 12th postoperative day.

**Fig. 3:**
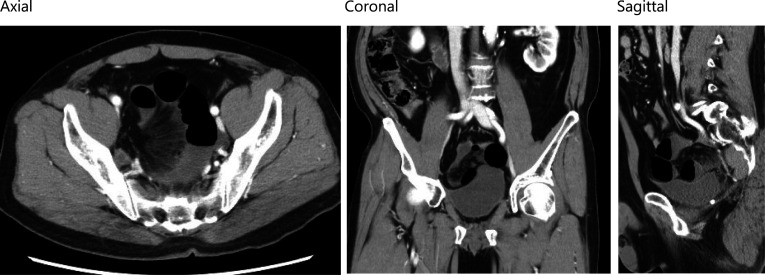
Contrast-enhanced CT scan of the case 2 showed closed-loop in the right pelvic cavity (left: axial, center: coronal, right: sagittal)

**Fig. 4:**
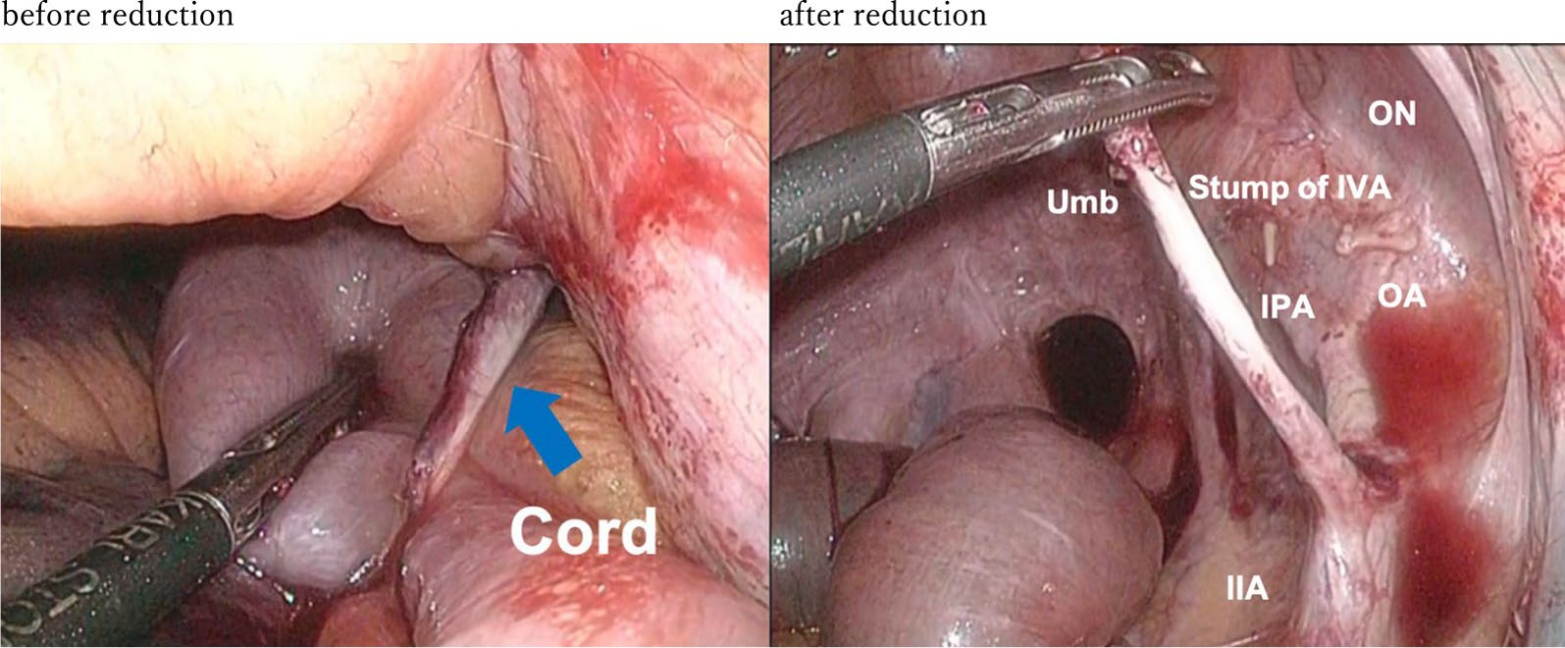
Laparoscopic finding in case 2. Left: small bowel herniating into the space after lateral lymph node dissection. Right: the hernial orifice created the umbilical artery cord (Umb). IIA: internal iliac artery, IPA: internal pudendal artery, IVA, inferior vesical artery, ON: obturator nerve, OA: obturator artery

Case2: A 57-year-old man visited our emergency room because of sudden onset of abdominal pain and vomiting. Eight months prior, he had undergone a robot-assisted rectal amputation with LLND following neoadjuvant chemotherapy for rectal cancer. The LLND was the same technique as in case 1. No anti-adhesion material was used. He subsequently received adjuvant chemotherapy with FOLFOX. No recurrent sign was observed. Contrast-enhanced CT images revealed that SBO with a closed-loop had occurred in the right pelvic cavity (Fig. 3). There was edema in the mesentery and a small amount of ascites, but intestinal blood flow was maintained. Contrast-enhanced CT did not show any clear ischemic findings in the intestine; and whereas there was tenderness in the upper abdomen, there were no notable findings in the lower abdomen. Therefore, the patient was admitted for observational management. The next day follow-up CT showed same findings as yesterday, however abdominal bloating was worsening. The patient was considered to have strangulated bowel obstruction caused by exposed vessels and nerves in the pelvis after LLND as in case 1.Therefore, we performed emergent laparoscopic surgery. The operation was started with 3 ports, but the dilated intestine could not be completely eliminated, and it was difficult to secure the operative field, so 4 ports operation was performed. We found the strangulated small bowel in the right pelvic with a band. Although congestion was observed in the strangulated small intestine, there were no signs suggesting necrosis based on its coloration. We released the strangulated small bowel by gentle manipulation. Results showed a band in the right pelvic region, which caused the internal hernia orifice of the strangulated small bowel. It became clear that the band constricting the internal hernia orifice was the right umbilical artery cord (Fig. 4). The venous congestion improved, and peristaltic movements in the strangulated intestine were immediately observed. Therefore, it was deemed feasible to preserve the intestine, and its resection was not performed. We resected the right umbilical artery cord to prevent further re-herniation. The patient progressed favorably after the operation. He was discharged with no major complication on the 7th postoperative day.

## Discussion

We experienced two cases of strangulated SBO after LLND in laparoscopic and robot-assisted surgery for rectal cancer. In both cases, emergent operations were necessary. The band in the first case was the left obturator nerve, whereas the other was the right umbilical cord. The two cases both had a closed-loop in the pelvic cavity, but with different symptoms. It was difficult to speculate on the cause of the SBO. Especially in the first case, the patient presented with atypical symptoms for a SBO, including severe pain in the left thigh without abdominal pain. Symptoms of the first case were thought to have a background of Howship–Romberg sign (HR sign). If we had had experience with cases of internal hernias after LLND, we might have been able to keep this possibility of internal hernias in mind and could have accurately assumed the pathogenesis of the hernia preoperatively.

The most common cause of SBO is surgical adhesions. Internal hernias account for approximately 0.5–5.8% of all cases of intestinal obstruction. More than 90% of all internal hernias are caused by natural or artificial orifices built by the intestine [1]. Internal hernias are an uncommon cause of an SBO, but it is more uncommon that an exposed blood vessel and nerve after pelvic lymphadenectomy (PL) form an internal hernia and cause of an SBO.

PL including LLND is one of the standard procedures performed for several malignant diseases, such as ovarian, cervical, endometrial, prostate, bladder, and rectal cancers [2–7]. We searched the PubMed database using the key words “internal hernia”, “pelvic lymphadenectomy (or lymph node dissection)” between 1978 and 2022. 21 cases have been described in 18 reports, including ours (Table 1) [8–24]. Strangulated internal hernia involving the right common iliac artery after PL in a patient with testicular cancer was first reported in 1978 [8]. No report for 30 years thereafter described strangulated internal hernia related to skeletonized vessels or nerves after PL. In 2008, Kim et al. reported strangulated internal hernia involving the right external iliac artery in a patient with cervical cancer [9]. Since then, case reports of PL-related strangulated SBO are increasing in recent years. Ten cases were reported in gynecology, six in urology, and three in gastrointestinal surgery. In previous reports, the external iliac artery and common iliac artery were the most common causes of SBO. In general, PL in gynecology and urogynecology involves an intensive dissection for the lymph nodes whole around the common iliac artery and external iliac artery [2, 3, 5]. On the other hand, these lymph node dissections can be omitted in rectal cancer [7]. Therefore, there are few reports in rectal cancer.

**Table 1:** Reported cases of strangulated small bowel obstruction after pelvic lymphadenectomy

Year	Patient	Cancer	Original approach	Duration	Hernia orifice	Treatment	Bowel resection	Orifice repair method
1978 [8]	52/M	Testicular	Open	4 months	Rt CIA	Open	Yes	Peritoneal closure
2008 [9]	67/F	Cervical	Lap	3 months	Rt EIA	Open	Yes	Peritoneal closure
2013 [10]	56/F	Ovarian	Lap	4 years	Lt EIA	Open	No	Unrepaired
2014 [11]	39/F	Cervical	Lap	2 years	Rt CIA	Lap to open	Yes	Coating with a collagen patch
2015 [12]	50/M	Bladder	Robot	5 months	Rt CIA	Open	Yes	Peritoneal closure
2016 [13]	50/M	Prostate	Robot	1 year	Rt CIA	Open	Yes	With collagen patch and peritoneal closure
2018 [14]	38/F	Cervical	Lap	6 months	Rt UA and ON	Lap	Yes	Resection of the UA
2018 [15]	68/M	Rectal	Lap	4 months	Rt SVA	N/A	Yes	Unrepaired
2018 [15]	59/M	Rectal	Lap	2 months	Rt SVA	Lap	Yes	Resection of the SVA
2018 [16]	64/M	Prostate	Robot	1 year	Rt EIA	Lap to open	Yes	Unrepaired
2019 [17]	72/M	Prostate	Robot	2 months	Rt EIA	Open	Yes	Unrepaired
2020 [18]	63/M	Rectal	Robot	1 month	Rt ON	Lap	Yes	Unrepaired
2020 [19]	78/M	Bladder	Lap	38 months	Rt ON	Open	Yes	Unrepaired
2020 [20]	68/F	Endometrial	Lap	7 years	Rt EIA and EIV	Open	Yes	Resection of the SVA
2020 [21]	53/F	Cervical	Lap	1 month	Rt SVA	Open	Yes	Resection of the SVA
2021 [22]	46/F	Cervical	Lap	9 years	Lt EIA	Lap to open	No	Peritoneal closure
2021 [23]	67/F	Ovarian	Open	6 years	Rt EIA/V	Lap	No	Resection of the Rt EIV
2022 [24]	57/F	Endometrial	Lap	9 months	Rt UA/ON	Lap to open	Yes	Resection of the Rt UA and ON
2022 [24]	62/F	Cervical	Lap	6 months	Rt UA	Lap to open	Yes	Resection of the Rt UA
2023	68/M	Rectal	Lap	52 months	Lt ON	Open	No	Unrepaired
2023	57/M	Rectal	Robot	8 months	Rt UA	Lap	No	Resection of the UA

Open: open surgery, Lap: laparoscopic surgery, Robot: robot-assisted surgery, Rt: right, Lt: left, CIA: common iliac artery, IA/V: external iliac artery/vein, UA: umbilical artery, SVA: superior vesical artery, ON: obturator nerve, N/A: not applicable

SBO caused by adhesions is significantly less common with laparoscopic surgery [25]. However, in our cases, the laparoscopic and robot-assisted surgery may have reduced adhesions and preserved the mobility of the intestinal tract, which may have facilitated the small intestine to fit into the gap formed by the nerves and blood vessels exposed by the lymph node dissection. Nine of ten cases previously reported in gynecology were postoperative laparoscopic cases. Except for the first report, in urology, five cases were performed laparoscopically or robot-assisted surgery. This may be due to the fact that postoperative adhesions are less likely to occur in these cases.

The 2014 JSCCR Guidelines for Treatment of Colorectal Cancer list total mesorectal excision (TME) or mesorectal excision (ME) with LLND as the standard procedure for lower rectal cancer in Japan [26]. In the JCOG0212 study, ME with LLND had a lower local recurrence, especially in the lateral pelvis, compared to ME alone [27]. As for the approach method, the usefulness of laparoscopic or robot-assisted rather than open approaches have been reported [28, 29]. In our hospital, LLND has been performed laparoscopically since 2013 and robot-assisted since 2018, and we experienced two cases of this disease. In previous reports, both cases of rectal cancer were performed laparoscopic or robot-assisted surgery [15, 18]. Although LLND is not standard in Western countries, technical improvements in minimally invasive surgery have resulted in rapid technical standardization of this complicated procedure [30]. Laparoscopic and robot-assisted approaches to LLND for rectal cancer are becoming ordinary, so it is very important to be aware of this disease.

In previous reports, most of the patients complained of abdominal symptoms, but when the obturator nerve forms an internal hernia and caused intestinal obstruction, some patients, including the case 1, complained of inguinal or thigh pain.[18, 24]. Therefore, it is necessary to be very careful when examining the patient with a history of PL.

In both of the two cases, we found characteristic findings in the pelvis on CT. The small intestine strangulated by internal hernia is seen in the pelvis, and the starting point of the intestinal obstruction is located in the lateral pelvic wall in both cases. Similar CT findings have been seen in several previous reports [15, 16, 24]. If a patient with a history of PL presents with symptoms of SBO, physical findings, or HR sign, and we find characteristic CT findings in the pelvis, we need to consider this disease as a SBO formed by an internal hernia with exposed vessels and nerves in the pelvis. As in the second case, there was a case reported in the past in which the diagnosis of internal hernia was not made, and it took a long time before surgery was performed [24]. If we diagnose this disease, the internal hernia is unlikely to be resolved, so we should perform surgery as soon as possible.

Based on our experience with only two cases of SBO due to an internal hernia in the pelvis, we can expect little or no intra-abdominal adhesions in cases of bowel obstruction. Therefore, a more minimally invasive laparoscopic approach should be considered. However, the second case could have been performed laparoscopically, the dilated small intestine obstructed the development of the surgical field, making surgical manipulation difficult in some situations. As a countermeasure, in order to secure the best possible surgical field and to perform the laparoscopic surgery more reliably, if there is no intestinal ischemia and there is time to spare before the surgery, a nasogastric tube or ileus tube should be implanted before the surgery to decompress the intestinal tract and reduce intestinal dilatation.

Prevention of internal hernias at the time of initial surgery is also an important issue, but there is no clear consensus on this issue. Prevention of internal hernia by peritoneal closure at the time of initial surgery or by covering with an artificial material may be considered. However, peritoneal closure may cause pelvic lymph fistula, and artificial covering may cause infection. So far there is no effective internal hernia prophylaxis at the time of initial surgery. Therefore, we believe it is important to keep this disease firmly in mind and to respond promptly and appropriately if a patient with this condition is seen.

## Conclusion

It is possible that the laparoscopic/robotic-assisted surgery has made the intestinal tract, which has less adhesions and preserved mobility, easier to fit into the gap formed by the exposed vessels and nerves after PL. Although cases of strangulated SBO due to an exposed intestinal cord after PL are rare, today, with the establishment of minimally invasive surgery, one may encounter this disease so it is important to keep this disease in mind.

## Data Availability

Not applicable.

## Abbreviations

LLND: Lateral lymph node dissection
SBO: Small bowel obstruction
PL: Pelvic lymphadenectomy
CT: Computed tomography
HR sign: Howship–Romberg sign
TME: Total mesorectal excision
ME: Mesorectal excision
